# Molecular Insights and Therapeutic Advances in Low-Risk Myelodysplastic Neoplasms: A Clinical Review

**DOI:** 10.3390/cancers17223610

**Published:** 2025-11-09

**Authors:** Vikram Dhillon, Jaroslaw Maciejewski, Suresh Kumar Balasubramanian

**Affiliations:** 1 Department of Oncology, Karmanos Cancer Institute, Wayne State University, Detroit, MI 48201, USA; hf9375@wayne.edu; 2 Department of Translational Hematology and Oncology Research, Taussig Cancer Institute, Cleveland Clinic Foundation, Cleveland, OH 44195, USA; maciejj@ccf.org

**Keywords:** Myelodysplastic neoplasms, molecular stratification, Inflammation and innate immune signaling

## Abstract

**Simple Summary:**

Myelodysplastic neoplasms are bone marrow disorders where blood cells fail to develop properly, causing low blood counts and requiring frequent transfusions. Patients with lower-risk MDS often survive for years but face significant morbidity and quality-of-life challenges. Recent advances in molecular profiling reveal that specific gene mutations may predict which treatments work best for individual patients. This review examines current and emerging therapies for lower-risk MDS, including drugs that improve red blood cell production (luspatercept, erythropoietin), drugs targeting genetic abnormalities (lenalidomide), and promising new agents that may slow disease progression. We discuss how molecular testing guides treatment selection and highlight inflammation’s role in disease development. Understanding these molecular features and matching them to appropriate therapies represents a shift toward personalized medicine, potentially transforming MDS from a chronic condition requiring lifelong management to one amenable to cure.

**Abstract:**

Myelodysplastic neoplasms (MDS) are characterized by remarkable heterogeneity in clinical manifestations, posing significant management challenges arising due to genetic plasticity. While the Revised International Prognostic Scoring System (IPSS-R) has traditionally stratified MDS into higher-risk (HR) and lower-risk (LR) categories, the recently developed Molecular International Prognostic Scoring System (IPSS-M) integrates molecular signatures and has further enhanced prognostic stratification. In LR-MDS, current therapeutic interventions remain non-curative and the goal of treatment is centered along three critical axes: reducing transfusion dependence, improving quality of life, and reducing the risk of progression to acute myeloid leukemia (AML). This review examines recent progress made in the therapeutic landscape of LR-MDS, with particular emphasis on the molecular basis of these novel agents that may have disease-modifying potential. We evaluate the clinical trials and targeted agents in the pipeline for treating LR-MDS, providing a comprehensive perspective where these treatment modalities are placed in the current standard of care and how these novel targets can shape future therapeutic innovations.

## 1. Introduction

**Figure 1:**
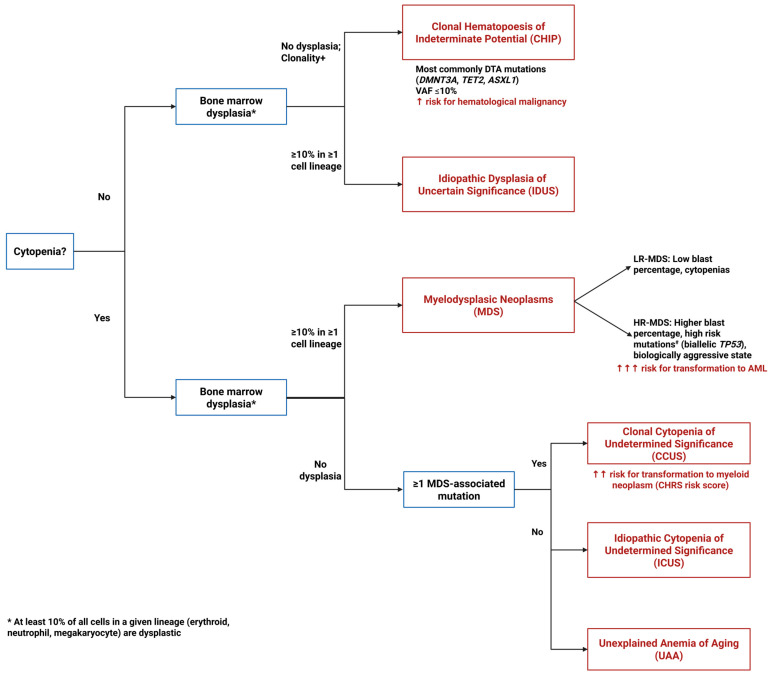
Diagnostic algorithm for pre-MDS conditions. This flowchart illustrates the diagnostic pathway for distinguishing between clonal and non-clonal cytopenias and dysplasia that can precede or may progress to MDS. The algorithm differentiates four key entities based on the presence or absence of cytopenias, dysplastic features, and clonal mutations: ICUS (Idiopathic Cytopenias of Uncertain Significance)—cytopenia without clonal mutations or dysplasia; CCUS (Clonal Cytopenias of Uncertain Significance)—cytopenia with clonal mutations but without dysplasia; CHIP (Clonal Hematopoiesis of Indeterminate Potential)—clonal mutations without cytopenia or dysplasia; and IDUS (Idiopathic Dysplasia of Unknown Significance)—morphologic dysplasia without cytopenia or clonal mutations. ^#^ High risk mutations include *ASXL1*, *CBL*, *DNMT3A*, *ETV6*, *EZH2*, *IDH2*, *KRAS*, *NPM1*, *NRAS*, *RUNX1*, *SF3B1*, *SRSF2*, and *U2AF1*.

Myelodysplastic neoplasms (MDS) represent a molecularly heterogeneous spectrum of clonal hematopoietic disorders characterized by ineffective hematopoiesis, peripheral cytopenias, and variable risk of leukemic transformation, occurring most commonly between the sixth and eighth decades of life [1]. While traditionally classified by morphologic features and cytogenetic abnormalities, the integration of comprehensive molecular profiling has fundamentally reshaped our understanding of disease biology and clinical behavior in MDS. The recent development of the International Prognostic Scoring System-Molecular (IPSS-M) formalized the critical role of somatic mutations in risk stratification, demonstrating that molecular signatures provide prognostic information independent of conventional clinical and cytogenetic parameters [2]. This molecular framework extends beyond traditional diagnostic boundaries, revealing a biological continuum encompassing Clonal Hematopoiesis of Indeterminate Potential (CHIP), Clonal Cytopenia of Unknown Significance (CCUS), Idiopathic Cytopenia of Unknown Significance (ICUS), and Idiopathic Dysplasia of Unknown Significance (IDUS) (Figure 1). Specific somatic alterations including del(5q), *SF3B1* mutations, *TP53* alterations, and other recurrent mutations not only stratify patients into distinct prognostic subgroups but increasingly serve as predictors of differential therapeutic responses.

**Figure 2:**
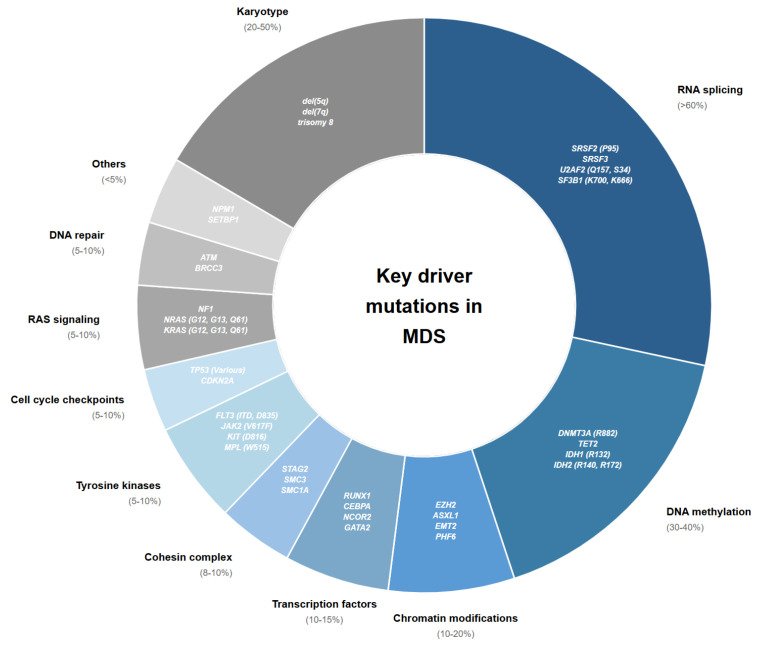
Key mutations and mutational classes with prognostic and therapeutic implications in MDS.

The therapeutic landscape for LR-MDS has expanded considerably over the past two decades, with treatment approaches ranging from supportive care and erythropoiesis-stimulating agents to immunomodulatory drugs, erythrocyte maturation agents (TGF-β inhibitors), and telomerase inhibitors (Figure 2). However, a critical challenge persists: the efficacy of these interventions varies substantially across molecular subgroups, and uniform increases in survival rates have been demonstrated. Despite responses in molecular signatures, validated biomarkers to guide treatment selection remain limited. Lenalidomide demonstrates exceptional activity in del(5q) LR-MDS with erythroid response rates exceeding 60% yet shows modest efficacy in non-del(5q) disease. Similarly, luspatercept achieves significantly higher response rates in *SF3B1*-mutated patients compared to *SF3B1* wild-type cohorts, while the impact of co-occurring mutations on treatment outcomes remains incompletely characterized [3]. Beyond specific mutational drivers, inflammatory signatures characterized by elevated IL-1β and IL-18 levels may influence clonal selection and disease progression, though how these patterns intersect with mutational profiles to determine therapeutic responsiveness requires further elucidation. As novel agents targeting specific molecular pathways advance through clinical development, identifying which molecular features predict treatment benefit, primary resistance, or risk of clonal evolution becomes imperative. This review synthesizes the current therapeutic armamentarium for LR-MDS through the lens of molecular stratification, examining how mutational landscapes influence treatment outcomes with approved therapies, analyzing emerging data linking molecular signatures to novel agent efficacy, identifying critical knowledge gaps where biomarker development could guide precision medicine, and also emphasizing where these molecularly targeting therapies should be repurposed for a curative potential, possibly in combination therapeutic strategies.

## 2. Methods

This narrative review synthesized evidence from PubMed/MEDLINE, Embase, and ClinicalTrials.gov from January 2005 through August 2025. Search terms included combinations of “myelodysplastic syndrome,” “lower-risk MDS,” “molecular signatures,” “SF3B1,” “TP53,” “del(5q),” “luspatercept,” “lenalidomide,” “imetelstat,” “erythropoiesis-stimulating agents,” “TGF-β inhibitors,” “inflammasome,” and “telomerase inhibitor.” We prioritized phase II/III clinical trials, prospective observational studies, mechanistic investigations, and recent comprehensive reviews. Relevant abstracts from major hematology conferences (ASH and EHA) from 2022 to 2024 were included to capture emerging trial data. Studies were selected based on clinical relevance to therapeutic decision-making in LR-MDS, with emphasis on molecularly stratified outcomes and novel disease-modifying agents.

## 3. Therapeutic Strategies in Low Risk-MDS

**Figure 3:**
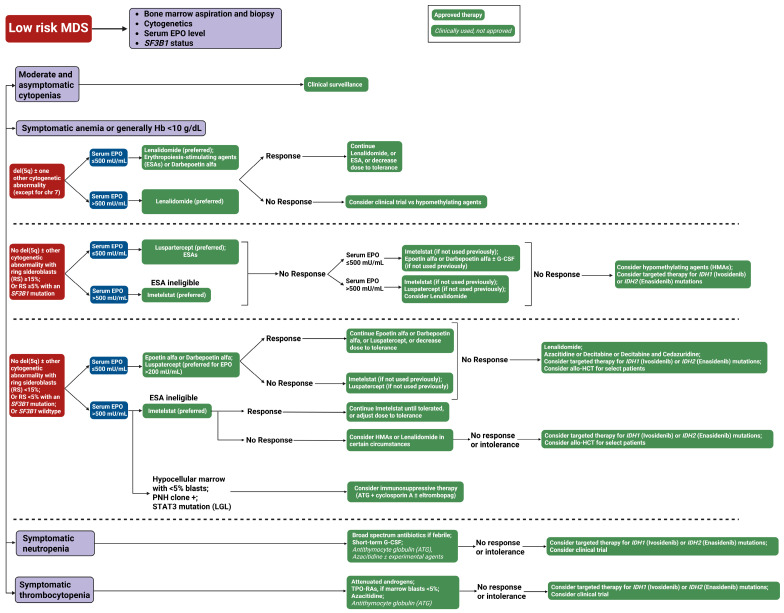
Our treatment approach to low-risk myelodysplastic syndrome (LR-MDS). This flowchart outlines the therapeutic approach to low-risk MDS based on clinical presentation and laboratory parameters. The algorithm stratifies patients into four categories: Moderate and asymptomatic cytopenias (observation only), symptomatic anemia, symptomatic neutropenia, and symptomatic thrombocytopenia. Green boxes with solid backgrounds indicate FDA-approved therapies; green boxes with italicized text denote clinically used but not formally approved treatments. Blue boxes represent clinical decision points. Abbreviations: ATG, antithymocyte globulin; del(5q), deletion of chromosome 5q; chr, chromosome; EPO, erythropoietin; G-CSF, granulocyte colony-stimulating factor; Hb, hemoglobin; MDS, myelodysplastic syndrome; RBC, red blood cell; RS, ring sideroblasts; SF3B1, splicing factor 3B subunit 1 gene; TPO-RAs, thrombopoietin receptor agonists; and U/L, units per liter.

Treatment in LR-MDS is personalized with the goal of improving quality of life and reducing morbidity associated with MDS. As a general approach to managing patients with LR-MDS, most therapeutic interventions focus on managing cytopenias, frequently addressing anemia, and reducing transfusion-associated complications [1] (Figure 3). We will highlight the therapies that are commonly used in the management of LR-MDS based on their mechanism of action. Details of the therapeutic agents and their pivotal clinical trials are summarized in Table 1.

### 3.1. Erythroid Maturation Agents and TGF-β Superfamily Signaling: Luspatercept

Transforming growth factor-beta (TGF-β) signaling is constitutively upregulated in LR-MDS and growth differentiation factors (GDFs) are expressed at higher levels compared to healthy individuals [2]. Luspatercept, a recombinant fusion protein consisting of a human activin receptor type-2B (ActRIIB) extracellular domain fused to an IgG Fc domain, functions as a ligand trap targeting GDF11 and other TGF-β superfamily ligands, thereby inhibiting SMAD2/3-dependent downstream signaling and rescuing late-stage erythropoiesis [3,4,5]. Similarly, Elritercept (KER-050) is a modified activin receptor type IIA ligand trap designed to bind and inhibit activin A and other select TGF-β superfamily ligands (GDF 8 and GDF11) [6]. Phase II data demonstrated a 55.2% overall erythroid response rate with a median TI duration of 134.1 weeks, showing efficacy in both ring sideroblast-positive and non-ring sideroblast patients, including those with high transfusion burden. The ongoing phase III RENEW trial (NCT06499285) is evaluating Elritercept in TD LR-MDS patients stratified by ring sideroblast status and baseline transfusion burden [7]. The proof-of-concept phase II PACE-MDS trial enrolled 108 patients irrespective of transfusion burden, ringed sideroblast (RS) status, or prior erythropoiesis-stimulating agent (ESA) exposure, establishing luspatercept’s broad therapeutic potential [8]. The study defined the molecular determinants of luspatercept response. Patients with *SF3B1* mutations (57% of the cohort) showed markedly higher hematologic improvement and erythroid rates (74.5%) compared with SF3B1 wild-type cases (38.8%). In contrast, mutations in other splicing factors (*SRSF2*, 11%; *U2AF1*) and DNA methylation genes (*TET2*, 39%; *DNMT3A*, 21%) conferred similar response rates regardless of mutational status. Notably, chromatin modifier mutations such as *ASXL1* (17%), typically linked to poor prognosis, also demonstrated favorable responses to luspatercept [8].

Based on the encouraging results from PACE-MDS, the phase III MEDALIST trial served as the pivotal registration trial for luspatercept in LR-MDS with RS [9,10]. The primary outcome of TI (transfusion independence) within 24 weeks was achieved in 38% of the luspatercept arm, with a 31-week median duration of response (DOR). Moreover, 93% of luspatercept-treated patients harbored SF3B1 mutations and response rates remained consistent regardless of SF3B1 allelic burden or total mutational burden. The phase III COMMANDS trial was a paradigm shift by comparing frontline luspatercept with epoetin alfa in 363 ESA-naive, TD LR-MDS patients irrespective of ring sideroblast status [11]. Molecular analyses demonstrated superior luspatercept responses across spliceosome mutations, with *SF3B1*, *SF3B1α* (co-mutated with *DNMT3A*, *ASXL1*, and/or *TET2*), and *U2AF1* variants all showing enhanced efficacy over epoetin alfa. *SF3B1α* co-mutants achieved the highest response rates. Importantly, luspatercept also demonstrated superior efficacy over epoetin alfa in SF3B1 wild-type patients, with sustained responses observed across both mutated and non-mutated cohorts. Favorable responses were also observed in DNA methylation (*TET2*, *DNMT3A*) and chromatin modifier (*ASXL1*, *EZH2*, and *IDH2*) mutations, independent of overall mutational burden [11]. A Japanese validation cohort reported 90% response rates in SF3B1-mutated, 62.5% in *TET2*-mutated, and 83.3% in *SF3B1*/*TET2* co-mutated patients [12]. Real-world data further identified *SF3B1*-mutated MDS with concurrent del(5q) to show inferior outcomes on hematologic improvement [13].

### 3.2. Erythropoiesis-Stimulating Agents (ESAs)

ESAs including epoetin and darbepoetin alfa represent first-line therapy for anemia in LR-MDS patients with low transfusion burden. Beyond the traditional prognostic factors such as serum EPO levels ≤ 500 U/L, low blast count (< 5%), very low-to-low IPSS scores, and absence of multilineage dysplasia, molecular profiling helps to refine our understanding of ESA responsiveness. [14]. Patients harboring del(5q) or demonstrating *STAT3* hyperphosphorylation exhibit diminished ESA responsiveness with characteristically partial and transient responses [14]. Splicing factor mutations show variable impact; *SF3B1* mutations demonstrate conflicting ESA response data, while cohesin complex mutations, specifically *STAG2* (an X-linked gene), are significantly associated with ESA resistance in male LR-MDS patients [15,16]. Flow cytometric profiling reveals that ESA responders exhibit a higher proportion of immature erythroid precursors (CD117+), suggesting that cytofluorimetric assessment of the bone marrow erythroid compartment may complement genomic profiling in therapeutic decision-making [17].

Registry-based analyses of EPOANE and ARCADE trials reveal that ESA responses typically manifest within 12 weeks, with a median duration of approximately two years, and response kinetics potentially influenced by clonal burden and mutational family composition [18,19,20]. Early ESA initiation within six months of diagnosis is thought to correlate with higher response rates and delayed RBC transfusion dependence; the ongoing phase III EPO-PRETAR trial (NCT03223961) will prospectively validate preemptive ESA strategies [21]. Given the mechanistic complementarity between ESAs (promoting early stage erythroid maturation) and luspatercept (rescuing late-stage maturation through TGF-β superfamily inhibition), combination therapy in 28 patients refractory to luspatercept monotherapy demonstrated a 36% overall HI rate [22]. Notably, 71% of patients with secondary luspatercept resistance responded to combination therapy, suggesting ESA-mediated resensitization may restore early progenitor pools or upregulate erythropoietin receptor density on late-stage progenitors [22].

### 3.3. Immunomodulatory Drugs (IMiDs): Lenalidomide

Patients with TD del(5q)-MDS demonstrate unique sensitivity to lenalidomide through a mechanism of synthetic lethality involving the haploinsufficiency of *CSNK1A1* (encoding CK1α), a serine/threonine kinase in the common deleted region of chromosome 5q. Lenalidomide promotes the proteasomal degradation of *CK1α*, and in the setting of CSNK1A1 haploinsufficiency, this induces selective cytotoxicity against del(5q) progenitors via E3 ubiquitination-mediated apoptosis [23]. Clinical validation of lenalidomide’s molecular selectivity emerged through pivotal trials (MDS-003 and MDS-004) demonstrating marked efficacy in del(5q) MDS, with transfusion independence achieved in 56–76% of patients harboring isolated 5q deletions [24,25,26], while non-del(5q) LR-MDS showed more modest responses (27% RBC-TI in ESA-refractory patients). Co-occurring mutations in *TP53*, *RUNX1*, or *CSNK1A1* define adverse molecular subsets [27]. *TP53* mutations occur in roughly 20% of lower-risk del(5q) cases and confer poor cytogenetic response, inferior survival, and increased risk of leukemic transformation, particularly when present as biallelic (multihit) lesions, which now reclassifies the disease as *TP53*-mutant MDS rather than true del(5q) [27]. Even monoallelic *TP53* variants with high VAF (>20%) portend adverse outcomes. Additional deleterious co-mutations include *RUNX1*, which strongly predicts progression and *CSNK1A1*, which is linked to lenalidomide resistance and shorter remission duration [27]. In contrast, DTA mutations (*DNMT3A*, *TET2*, and *ASXL1*) often reflect age-related clonal hematopoiesis and appear to exert a limited independent prognostic impact [27]. Even in non-transfusion-dependent patients, the phase III SintraREV trial demonstrated that early use of low-dose lenalidomide in non-transfusion-dependent del(5q) patients delayed the time to transfusion dependency [28]. The majority of del5q MDS patients eventually develop resistance to lenalidomide, prompting the investigation of PP2A as next-line therapy after lenalidomide failure [29]. LB-100 is a direct inhibitor of PP2A, currently undergoing clinical development in phase I/II trials (NCT03886662) where 47 LR-MDS patients will be treated with the drug.

### 3.4. Thrombopoietin Mimetics

Thrombopoietin receptor agonists (TPO-RAs) activate *JAK2*/*STAT5*, *MAPK*, and *PI3K*-*AKT* signaling pathways to promote megakaryocytic differentiation, extend platelet lifespan, and reduce apoptotic [30,31]. Second-generation TPO-RAs (romiplostim, eltrombopag, and avatrombopag) that overcome the immunogenicity of first-generation agents that generated neutralizing antibodies cross-reactive with endogenous TPO Splicing factor mutations are critical determinants of TPO-RA responsiveness in LR-MDS with thrombocytopenia; SRSF2 mutations significantly predict romiplostim response, occurring in 41% of responders versus 16% of non-responders, with patients harboring SRSF2 mutations achieving 65% response rates compared to 33% in wild-type patients [32]. Broader spliceosome mutations (*SRSF2*, *SF3B1*, *U2AF1*, and *ZRSR2*) collectively associate with response, present in 67% of responders versus 35% of non-responders, suggesting that aberrant splicing machinery may render megakaryocyte progenitors particularly sensitive to TPO receptor stimulation [33].

Eltrombopag demonstrates dual mechanisms beyond TPO receptor activation: iron chelation that reduces labile iron-induced ROS damage and restoration of iron homeostasis, particularly relevant in RS-associated MDS [34]. The clinical efficacy of eltrombopag was assessed in the phase II EQoL-MDS trial, with 42% platelet response rates with durable responses (median 340 days) and notable transfusion independence in 54% of treated patients, without increased AML transformation compared to placebo [35,36]. Combination strategies with hypomethylating agents (azacitidine; ELASTIC study) or lenalidomide are being carried out based on TPO-RAs’ ability to counteract myelosuppressive toxicities while preserving therapeutic efficacy. This is particularly relevant given lenalidomide’s propensity to induce significant thrombocytopenia in non-del(5q) LR-MDS patients. Prospective validation of these combinations will shed light on whether TPO-RAs can enhance the disease-modifying activity of existing agents [37].

### 3.5. Hypomethylating Agents (HMAs)

Hypomethylating agents (azacitidine or decitabine) are the standard of care for high-risk MDS but also alleviate cytopenia in lower-risk disease. Clonal *TET2* mutations (variant allele frequency > 10%) predict favorable HMA response, particularly in the absence of concurrent *ASXL1* mutations (OR 3.65), while *ASXL1* mutations are associated with decreased complete remission rates and patients with ≥3 driver mutations demonstrate significantly lower response rates [38]. Treatment with azacitidine for 5 days resulted in transfusion independence in half of transfusion-dependent patients, while abbreviated 3-day schedules have yielded promising results [39,40]. In one study, decitabine achieved a 67% transfusion independence rate compared to azacitidine (48%) (NCT01720225) [41]. On the other hand, oral azacitidine (CC-486) has demonstrated a 31% RBC-transfusion independence rate with 11-month median duration of response in a phase III trial (NCT01566695) [42].

Primary or secondary HMA failure is not uncommon, with median OS of 17 months in lower-risk disease post-failure. Aside from targeted therapy, allogeneic SCT remains the only curative approach, though median OS after alloHSCT in HMA-refractory patients is 39 months with 3-year relapse probability exceeding 50% [43]. In transplant-ineligible patients, oral formulations including decitabine/cedazuridine (ASTX727) are being investigated for LR-MDS (NCT02103478, NCT03502668), with cedazuridine also assessed in combination with azacitidine (NCT04608110) [44,45]. These fully oral regimens will decrease administration burden, improving quality of life for patients and caregivers.

### 3.6. Telomerase Inhibitor: Imetelstat

Imetelstat, a 13-mer oligonucleotide complementary to the RNA-template region of telomerase, competitively inhibits telomerase enzymatic activity that is upregulated in MDS cells [46]. In a pilot study on myelofibrosis, 77 patients treated with imetelstat achieved 21% complete or partial remission, with complete molecular remission accompanied by bone marrow fibrosis reversal in all responders, supporting disease-modifying activity. [47]. In this study, complete response rates were significantly higher in patients harboring *SF3B1* or *U2AF1* mutations (38%) compared to those without these spliceosome mutations (4%), while *ASXL1*-mutant patients demonstrated no responses (0%) versus 32% in ASXL1 wild-type patients, suggesting that spliceosome mutations may contribute to vulnerability through altered telomerase regulation [47].

Follow-up phase-II studies demonstrated durable transfusion independence in 42% of LR-MDS patients ineligible for ESA therapy [48]. The Phase-III IMerge trial (NCT02598661) enrolled 178 patients randomized to imetelstat or placebo [49]. In the treatment arm, about 40% achieved RBC-transfusion independence with durable responses lasting 52 weeks. Molecular profiling revealed *SF3B1* as the most frequently mutated gene (75.8%), with *SF3B1*-mutant patients demonstrating superior transfusion independence rates with Imetelstat versus placebo (48.8% vs. 16.3% at 8 weeks) [49]. Responses varied across *SF3B1* hotspots, with T663P and A744P mutations achieving 100% response rates, while the most common K700E hotspot achieved 43.9% response. Other frequently mutated genes also responded: *TET2*-mutant patients achieved 50% transfusion independence versus 21.4% with placebo, and *ASXL1*-mutant patients achieved 27.8% response versus 0% with placebo [49]. Notably, higher mutational burden (>2 mutations) was associated with enhanced response rates (45.5% versus 6.7% with placebo), and even patients harboring traditionally poor-prognosis mutations (*TP53*, *ETV6*, *RUNX1*, *ASXL1*, or *EZH2*) achieved 31.8% transfusion independence with Imetelstat versus 0% with placebo [50]. Neutropenia was the most serious adverse event (91% treatment arm vs. 47% placebo), though it is manageable with dose delays and reductions, and based on this trial, Imetelstat has received FDA approval for LR-MDS in heavily TD patients.

## 4. Experimental Therapies in the Pipeline for Low-Risk MDS

### 4.1. Roxadustat

Roxadustat is a hypoxia-inducible factor prolyl hydroxylase (HIF-PH) inhibitor and offers mechanistic advantages over ESAs; it stimulates endogenous EPO production while simultaneously reducing hepcidin to enhance iron bioavailability [51,52]. This dual mechanism suggested potential efficacy in ESA-refractory patients, a hypothesis supported by early phase data showing 37% TI rates independent of ring sideroblast status or baseline EPO levels [53]. Despite the promising TI rates in early phase trials, the phase III MATTERHORN trial failed to meet its primary endpoint and recapitulated these findings [54]. Three factors that may have contributed to the lack of TI are an unexpectedly high placebo response (33%), the inclusion of minimally transfusion-dependent patients (1 unit/8 weeks) with better natural history, and premature discontinuations in the roxadustat arm. Although post hoc analysis from the trial revealed roxadustat’s benefit concentrated in heavily transfused patients (≥2 units/4 weeks: 36% vs. 11% placebo), a pattern opposite to luspatercept, which performs better in lower transfusion burden patients [54]. A revised phase III trial focusing on ESA-refractory patients with high transfusion burden will start enrolling in late 2025. Additionally, rational combination strategies are being explored, including roxadustat plus luspatercept (NCT06006949) with complementary mechanisms of EPO stimulation and terminal erythroid maturation.

### 4.2. Interleukin-1 Receptor-Associated Kinases (IRAKs) and the NLRP3-Inflammasome Pathway

Toll-like receptors (TLRs) and their downstream mediator, MyD88, are overexpressed in LR-MDS, with TLR2 expression the highest in LR-MDS patients and *MyD88* overexpression detected in 40% of cases, correlating with shorter survival, providing strong rationale for targeting this pathway [55]. Mutations in *U2AF1* and *SF3B1*, present in over 50% of MDS—drive the expression of hyperactive *IRAK4* isoforms through distinct exon-retention mechanisms: *U2AF1* mutations induce exon 4 retention while *SF3B1* mutations cause exon 6 retention, both producing oncogenic IRAK4-Long (IRAK4-L) that assembles with *MyD88* into a hyperactive myddosome complex, driving maximal NF-κB and MAPK activation and sustaining malignant clone dominance [56]. While all U2AF1-mutant cases express *IRAK4-L*, approximately 50% of splicing factor wild-type cases also exhibit this isoform, with *IRAK4-L* expression correlating specifically with U2AF1 but no other splicing factor mutations like *SRSF2* [56]. *IRAK4* activation also represents an escape mechanism for therapeutic resistance, with *IRAK4* upregulated during *FLT3* inhibitor therapy through increased TLR9 expression, driving adaptive resistance in *FLT3*-mutant disease, providing rationale for dual *IRAK4/FLT3* inhibition even in diseases where *FLT3* mutations are less common [57]. Limited single-agent activity of selective *IRAK4* inhibitors revealed functional complementation by its paralog *IRAK1* through noncanonical, MyD88-independent pathways, with dual *IRAK1/4* inhibitors demonstrated superior suppression of disease-propagating cells and the induction of differentiation [58]. R289, a selective dual-*IRAK1/4* inhibitor, is being evaluated in heavily pretreated LR-MDS, achieving hematologic responses in 40% of transfusion-dependent patients treated with ≥500 mg once daily in a phase Ib trial (NCT05308264) [59]. Patients with spliceosome mutations demonstrate preferential responses to *IRAK* inhibition, with three of five evaluable patients achieving complete responses and these mutations create targetable dependency on *IRAK4-L* for disease-initiating cell functions, establishing splicing factor status as a predictive biomarker [60,61]. Clinical evaluation of emavusertib (dual *IRAK4*/*FLT3* inhibitor) combined with azacitidine or venetoclax is ongoing in LR-MDS cohorts (NCT04278768), though emavusertib monotherapy in LR-MDS (LUCAS trial, NCT05178342) was terminated early due to safety concerns. Both *IRAK1* and *IRAK4* are essential for NLRP3 inflammasome-mediated pyroptosis, which drives bone marrow inflammation in MDS, and direct inflammasome inhibition offers an alternative approach: HT-6184 (targeting NEK7, essential for NLRP3 assembly) showed promising transfusion independence in phase I (NCT05447546) and is advancing to phase I in LR-MDS, while DFV890 (direct NLRP3 inhibitor) is under phase Ib evaluation (NCT05552469) [62,63,64,65,66]. The optimal sequencing of therapies targeting the IRAK–inflammasome axis with current standards such as ESAs and luspatercept in LR-MDS remains to be defined through ongoing clinical trials.

### 4.3. Interleukin-Family Inhibitors: SX-682, Canakinumab, and BMS-986253

The IL-8/CXCR2 axis is a novel therapeutic target in LR-MDS, with CXCR2 overexpression predicting transfusion dependence and worse overall survival through the promotion of leukemic stem cell proliferation and myeloid-derived suppressor cell (MDSC)-mediated immunosuppression [67,68]. SX-682, an oral dual CXCR1/2 inhibitor, demonstrated dose-dependent efficacy in HMA-refractory patients, achieving a 50% overall response rate with reduction in marrow MDSCs and LSCs [69]. The ongoing phase II expansion (NCT04245397) evaluates SX-682 both as monotherapy and combined with decitabine/cedazuridine in HMA-naive and HMA-failure cohorts, based on evidence implicating CXCR2 signaling in HMA resistance. A direct IL-8 blockade is also being pursued with BMS-986253, an anti-IL-8 monoclonal antibody that attenuates aberrant erythroid proliferation and is being tested as monotherapy and in combination with decitabine/cedazuridine in phase I/II trials (NCT05148234). Additionally, talacotuzumab (JNJ-56022473), an anti-CD123 antibody targeting the IL-3 receptor α-subunit on leukemic stem cells, is being evaluated in combination with daratumumab in a phase II trial (NCT03011034) with primary endpoints of tolerability and TI.

Canakinumab, an anti-IL-1β monoclonal antibody targeting inflammasome activation, has revealed a critical principle in MDS therapeutics: efficacy is restricted to patients with lower genetic complexity, with single-cell RNA sequencing demonstrating that canakinumab rescued ineffective erythropoiesis only in patients harboring single-driver mutations in *TET2* or *DNMT3A* [70]. In a phase II trial of 25 heavily pretreated LR-MDS patients (80% HMA-failure), the overall response rate was 17.4%, with sustained transfusion independence > 12 months observed exclusively in patients with low clonal complexity [70]. A phase Ib/II combination study with darbepoetin alfa confirmed on-target inflammasome inhibition through a reduction in ASC specks but yielded no responses in this heavily pretreated population. The study has been redesigned to focus on patients with lower transfusion burden and *TET2*/*DNMT3A* mutations (NCT04798339) [71]. Recognizing that multi-pathway targeting may overcome resistance in genetically complex disease, a phase Ib trial (NCT04810611) is evaluating a triplet immunomodulatory regimen combining canakinumab with NIS793 (a TGF-β1 inhibitor) and sabotolimab (a TIM-3 inhibitor specific to leukemic stem cells and blasts) with a primary endpoint of safety. These evolving data suggest that IL-1β blockade may be most effective in earlier disease stages such as CCUS or genetically simple MDS, while CXCR2 inhibition may address stem cell-intrinsic mechanisms relevant across disease stages, including HMA-refractory disease [72].

### 4.4. Anti CD-33 Antibodies and Bi-Specific Immune Therapies

Recent evidence demonstrates that MDSCs accumulate more densely in the bone marrow of MDS patients, with significantly lower populations observed in very low/low-risk patients compared to intermediate/high-risk disease [73]. The CD33-S100A9 interaction promotes MDSC expansion and induces secretion of immunosuppressive cytokines (IL-10 and TGF-β) while generating reactive oxygen species that contribute to genomic instability, making it a key target in reversing immunosuppression in the MDS microenvironment [74]. The first-generation anti-CD33 monoclonal antibody BI-836858 was tested in 27 LR-MDS patients but was terminated due to lack of clinical activity. Importantly, treatment with BI-836858 failed to reduce CD33 expression in bone marrow or activate NK (NCT02240706). JNJ-67571244, a C2 domain-binding CD33 × CD3 bispecific antibody engineered to bypass CD33 polymorphic variability, demonstrated initial activity in relapsed/refractory AML and MDS but was discontinued in phase I due to dose-limiting cytokine release syndrome and hepatotoxicity. AMV564, another CD33/CD3 bispecific with an optimized structural design and improved pharmacokinetics, remains under clinical evaluation in higher-risk MDS. Emerging platforms such as second-generation nanobody-based TriKEs (GTB-3650) aim to couple NK-cell activation with myeloid-derived suppressor cell depletion, with clinical entry anticipated in 2025 [75,76]

### 4.5. RAS Pathway Inhibitor: Rigosertib

Oral rigosertib, a RAS-mimetic small molecule that targets *RAS* effector pathways and induces mitotic arrest, demonstrated encouraging activity in transfusion-dependent LR-MDS patients refractory to erythropoiesis-stimulating agents in early phase studies [77]. Upon treatment with rigosertib, myeloblasts experience mitotic stress due to a degradation product of rigosertib binding between α- and β-tubulin cells. This prevents microtubule growth, leading to apoptosis and a reduction in clone size, producing a clinical response [78]. The phase II ONTARGET trial evaluated oral rigosertib in TD LR-MDS patients who did not respond to ESA therapy and discovered that a dose of 560 mg resulted in a 44% TI rate [79]. Exploratory biomarker analysis identified a distinct genomic methylation profile associated with complete responders, characterized by hypermethylation of genes involved in transcription regulation, cell adhesion, and proliferation, suggesting potential for the preselection of patients likely to benefit. Phase III studies are underway to evaluate Rigosertib with azacitidine after ESA failure.

### 4.6. Spliceosome Modulation with SF3B1, PRMT5 and ATR Inhibitors

*SF3B1* mutations are prognostically favorable and rarely progress to AML, whereas *SRSF2* or *U2AF1* mutations are associated with worse overall survival [80]. H3B-8800, an *SF3B1* modulator, disrupts abnormal mRNA splicing, halting tumor cell proliferation, and the induction of apoptosis in *SF3B1*-mutant leukemia cells [81]. Encore-MDS (NCT02841540), a phase I/II trial, enrolled 84 patients (including 21 with LR-MDS) who were treated with the SF3B1-modulator H3B-8800. The drug demonstrated a favorable safety profile, but only a tenth of the cohort achieved TI. An interim analysis did not meet prespecified outcomes, leading to the discontinuation of drug development. Novel splicing modulators targeting the spliceosome complex formation and inducing alternative splicing such as *PRMT5* inhibitors (GSK3326595 and JNJ-64619178) and ceralasertib, an ATR inhibitor, are being investigated in LR-MDS for targeting the pre-mRNA splicing complex. Phase I dose tolerability studies are underway (NCT03770429, NCT03573310, and NCT03614728) for patients post-HMA-failure.

### 4.7. Whole Cancer Vaccine: K562/GM-CSF

Whole tumor vaccines deliver an antigenic payload that promotes T-cell clonal expansion to target cancer cells. GVAX is a whole-cell vaccine platform that uses engineered tumor cell lines to produce GM-CSF and induce an anti-tumor immune response [82]. A phase I study engineered a K562 cell line to express GM-CSF and induce anti-tumor immunity in three LR-MDS patients [83]. Although the primary endpoint was safety, the investigators also noted reduced transfusion requirements. Two novel tumor-associated antigens are being explored in the cancer vaccine platforms WT1 and PR1. WT4869 is a synthetic peptide vaccine derived from the WT1 antigen and was recently evaluated in a phase I/II study (JapicCTI-101374) involving 26 MDS patients, 9 of whom had LR-MDS [84]. The vaccine was well tolerated, and one-fifth of the patients exhibited a hematological response. As a proof-of-concept, the trial demonstrated leukemia-specific immunity derived from WT1 (NCT00270452). PR1 is a nine-amino-acid peptide present on the surface of myeloid leukemic cells and recognized by cytotoxic T lymphocytes when bound to HLA-A2 [85]. A PR1-based peptide vaccine was evaluated in a phase II setting (NCT00893997) for myeloid malignancies and PR1-specific immune response was seen in more than half of the patients. Phase II tumor vaccine trials focused on LR-MDS are currently underway (NCT00893997 and NCT00513578). Future tumor vaccine trials will incorporate novel platforms such as personalized mRNA vaccines and new mutation-specific targets.

### 4.8. Pexmetinib (ARRY-614) and CHRM4 Inhibitors

Abnormal activation of innate immune signaling cascades in LR-MDS leads to upregulation of p38 MAPK and an unregulated cytokine response. Apoptosis of the progenitor cells follows, resulting in ineffective hematopoiesis. ARRY-614 is an oral dual-inhibitor of p38 MAPK and Tie2 (a receptor tyrosine kinase necessary for maintaining HSPC self-renewal) that demonstrated good tolerability in a phase I setting (NCT00916227); 31% of LR-MDS patients demonstrated a hematological response per IWG-criteria [86]. Phase II studies are being planned to further assess efficacy.

Within the erythroid lineage, the burst-forming unit erythroid (BFU-E) represents the earliest lineage-committed progenitor cell. Pre-clinical work has identified that cholinergic receptor muscarinic 4 (*CHRM4*) is expressed on early erythroid progenitors and plays a crucial role in regulating BFU-E differentiation. Pharmacological inhibition of *CHRM4* using selective antagonists results in increased erythrocyte production, corrects anemia, and reduces hemolysis in murine models of MDS. Notably, *CHRM4* inhibition successfully rescued BFU-E functionality and reduced abnormal EPO levels to those comparable with wild-type mice, suggesting that *CHRM4* inhibition effectively overcomes EPO resistance in MDS. These promising pre-clinical findings are paving the way for the pre-clinical development of *CHRM4* inhibitors for LR-MDS.

### 4.9. Pyruvate Kinase Activators: Tebapivat (AG-946)

RBC-specific pyruvate kinase (PK) is thought to be maladaptive in LR-MDS. Tebapivat is an allosteric activator of PK isoforms, designed to increase glycolysis by rescuing PR function, which in turn improves RBC survival [87]. In a proof-of-concept phase I study (NCT04536792), a daily dose of 5 mg Tebapivat demonstrated safety and pharmacodynamic activity. Therefore, a stepwise phase II study (NCT05490446) with an expanded patient cohort is underway. The study will expand to recruit 96 LR-MDS patients with a primary endpoint of hemoglobin response per IWG criteria.

### 4.10. Ezatiostat (TLK199)

Ezatiostat is a synthetic glutathione-analog that functions as a reversible inhibitor of glutathione S-transferase P1-1 (GSTP1-1). Pre-clinical studies suggest that ezatiostat can promote the maturation of hematopoietic progenitors and increase ROS production in dysplastic cells, leading to the apoptosis of leukemic blasts [88]. In a phase I trial, 45 patients with LR-MDS were given ezatiostat and tolerated it safely (NCT00280631). In a follow-up phase II trial, ezatiostat was evaluated in 89 heavily pre-treated LR-MDS patients and demonstrated a hematological response in 29% of RBC-TD patients [89]. Interestingly, the subset of patients previously treated with lenalidomide alone had a TI rate of 40% suggesting a potential rescue strategy with ezatiostat [90]. An ezatiostat and lenalidomide combination was evaluated in a phase I trial (NCT01062152), enrolling 19 LR-MDS patients without del 5q. Overall, a hematological response was observed in 40% of patients, with 43% of RBC-TD and 60% of platelet-TD patients achieving TI.

### 4.11. RNA Therapies: SLN124

SLN124 is a conjugated 19-mer small interfering RNA (siRNA) engineered to target the *TMPRSS6* gene and alter hepcidin metabolism [91]. Hepcidin synthesis is negatively regulated by *TMPRSS6*. By silencing *TMPRSS6* in the liver, SLN124 increases endogenous hepcidin production, thereby restoring plasma iron homeostasis [91]. Gemini II (NCT04718844), a phase I study, is underway to evaluate the safety and tolerability of SLN124 in seven cohorts of 56 LR-MDS patients. The primary outcome is safety and key secondary outcomes include measurement of hepcidin and serum iron levels. If the safety outcomes are met, phase II trials will focus on whether reducing labile plasma iron levels alleviate anemia and improve erythroid hematopoiesis in LR-MDS.

### 4.12. TGF-β Signaling and ALK Inhibitors: Vactosertib and TP-0184

Constitutional activation of TGF-β signaling via SMAD2/3 leads to ineffective hematopoiesis in LR-MDS [92]. Dimerization of *ALK5* (a TGF-β receptor-type I) and TβRII (a TGF-β receptor-type II) defines the initiating event of the signaling cascade, known as the canonical signaling pathway [92]. Galunisertib, an oral inhibitor of the canonical signaling pathway, underwent a phase I trial (NCT01965808), and demonstrated a favorable side-effect profile. However, the drug development was discontinued for unknown reasons [93]. A newer canonical pathway inhibitor, vactosertib (TWEW-7197), inhibits the activity of TGF-β receptor-type I and suppresses the canonical signaling pathway, which in turn reduces levels of SMAD2, a key contributor to the ineffective hematopoiesis in MDS. A phase I/II trial (NCT03074006) to evaluate the safety of vactosertib showed a favorable safety profile, and phase II studies are underway, focusing on hematological improvement.

TP-0184, a dual *ALK2* and *ALK5* inhibitor, is currently in clinical development. A phase I/II study (NCT04623996) is being proposed to evaluate the safety and efficacy in 30 LR-MDS patients, followed by a cohort expansion of up to 60 patients in phase II [94]. These ongoing studies will shed light on the clinical benefits associated with the dual blockade of *ALK2* and *ALK5* in TGF-β biology.

**Figure 4:**
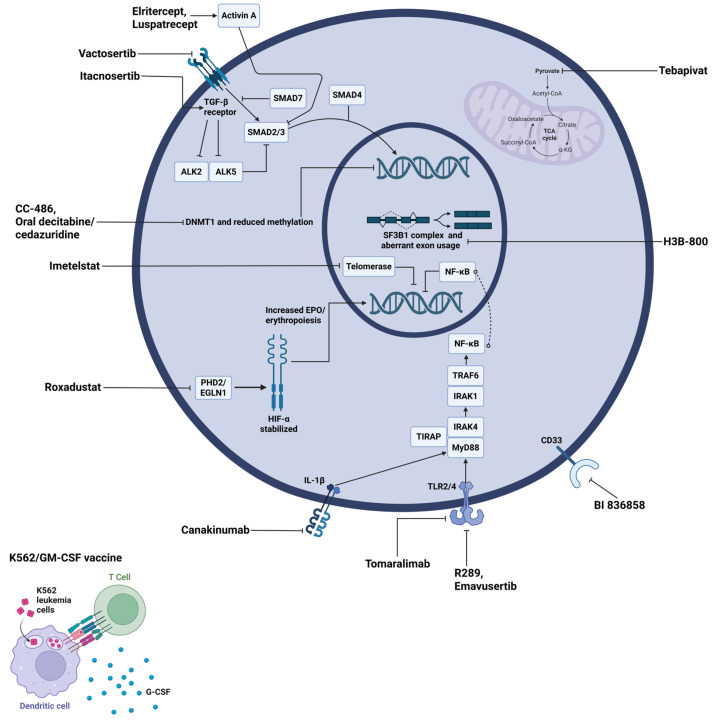
Molecular targets and therapeutic agents in LR-MDS. The diagram shows a representative MDS cell with its nucleus (inner circle) and mitochondrion (purple organelle). Therapeutic agents are labeled in black text with lines indicating their specific molecular targets. Abbreviations: ALK, activin receptor-like kinase; CoA, coenzyme A; DNMT1, DNA methyltransferase 1; EGLN1, egl-9 family hypoxia-inducible factor 1; EPO, erythropoietin; G-CSF, granulocyte colony-stimulating factor; GM-CSF, granulocyte-macrophage colony-stimulating factor; HIF-α, hypoxia-inducible factor alpha; IL-1β, interleukin-1 beta; IRAK, interleukin-1 receptor-associated kinase; MyD88, myeloid differentiation primary response 88; NF-κB, nuclear factor kappa B; PHD2, prolyl hydroxylase domain-containing protein 2; SF3B1, splicing factor 3B subunit 1; SMAD, mothers against decapentaplegic homolog; TCA, tricarboxylic acid; TGF-β, transforming growth factor beta; TIRAP, toll-interleukin 1 receptor domain containing adaptor protein; TLR, toll-like receptor; TRAF6, tumor necrosis factor receptor-associated factor 6; α-KG, alpha-ketoglutarate.

The molecular targets of current and investigational MDS therapies are summarized below in Figure 4, and emerging data from ongoing trials are summarized in Table 2.

## 5. Discussion

The therapeutic landscape of LR-MDS has expanded considerably with molecular profiling now guiding treatment selection and novel agents demonstrating unique mechanisms and possibly disease-modifying potential. Some of these molecular alterations can predict therapeutic sensitivity across multiple contexts: *SF3B1* mutations predict superior luspatercept efficacy, *SRSF2* mutations predict enhanced TPO-mimetic responsiveness, and *STAG2* mutations confer ESA resistance in males [16]. However, current clinical strategies mostly only capture mutational snapshots, missing dynamic clonal architecture, co-mutational contexts, and functional states that can influence therapeutic vulnerabilities.

Inflammation has emerged as a central pathogenic mechanism in LR-MDS in the last decade. The ImmunAID and EU-MDS consortium studies demonstrated that ASC/NLRP3 inflammasome activation in LR-MDS patients reaches levels comparable to autoinflammatory disorders, with TNF levels predicting disease progression and IL-6/IL-1β correlating with transfusion requirements [96]. Spliceosome mutations drive this inflammatory state through the mis-splicing of *IRAK4*, *CASP8*, and *MAP3K* genes, activating NF-κB pathways that simultaneously promote malignant clone growth while suppressing normal hematopoiesis. The identification of distinct immune subtypes, hyperactive versus moderate immune clusters, suggests that treating all LR-MDS patients uniformly with anti-inflammatory strategies may be suboptimal [96].

Rational combination strategies represent the logical next step, given that single-agent therapies face inevitable resistance through clonal selection and pathway redundancy. The mechanistic rationale for combinations like a TGF-β blockade plus an ESA (addressing both late-stage maturation arrest and early progenitor expansion) or *IRAK* inhibitors plus hypomethylating agents (targeting inflammation and epigenetic dysregulation simultaneously) is sound. Emavusertib’s preferential activity in spliceosome-mutant MDS, where aberrant splicing drives IRAK4-L overexpression, exemplifies how molecular context could guide combination selection. However, the history of failed HMA combination trials, where seemingly rational pairings showed no benefit or even harm, underscores that pre-clinical synergy does not guarantee clinical success. The emergence of disease-modifying agents, particularly imetelstat’s demonstration of sustained responses and variant allele frequency reduction, challenges the therapeutic nihilism that has long characterized LR-MDS management. Optimal sequencing, dosing schedules to avoid antagonistic cell-cycle effects, and patient selection based on pathway activation rather than mutation status alone will be critical.

The technological frontier with single-cell sequencing, multi-omic integration, and machine learning algorithms hold genuine promise for decoding response heterogeneity and guiding precision therapy [97]. Single-cell approaches could distinguish clonal competition (responsive populations overwhelmed by resistant clones) from uniform resistance, identifying therapeutic windows for combination intervention [98]. However, these remain as research tools that are not primetime for routine practice yet. Ultimately, transforming LR-MDS from a chronic condition managed palliatively to a potentially curable disease requires validated biomarkers that can predict not just prognosis but treatment-specific benefits.

## 6. Conclusions

LR-MDS has transitioned from supportive care to molecularly guided precision medicine. Molecular profiling using SF3B1, TP53, del(5q), and other mutations now directs therapeutic selection between luspatercept, lenalidomide, and emerging targeted agents. The ImmunAID consortium’s demonstration of inflammasome hyperactivation comparable to autoinflammatory diseases establishes inflammation as a targetable driver rather than consequence of dysplasia. Disease-modifying agents including telomerase and IRAK inhibitors offer potential for durable clone suppression beyond symptomatic improvement. However, translating mechanistic insights into clinical practice requires developing validated predictive biomarkers, optimizing rational combinations through rigorous clinical testing, and establishing intermediate molecular endpoints. Single-cell and multi-omic technologies promise to decode response heterogeneity but demand practical clinical surrogates. The convergence of molecular stratification and targeted therapeutics positions LR-MDS for transformation from palliation to disease modification, with a cure emerging as a tangible objective for molecularly defined populations.

## Figures and Tables

**Table 1:** Currently approved for low-risk MDS.

Agent	Class/Target	Included Patients	*N*	Efficacy Outcomes	Trial (Phase)
**Erythropoietin alpha,** **Darbepoetin alpha**	ESA	LR-MDS patients with anemia, low transfusion burden ^1^	130	ER: 45.9% vs. 4.4% (placebo)	EPOANE3021 (Phase III) ^2^
**Lenalidomide**	IMiDs	TD LR-MDS with del (5Q)	205	RBC-TI: 42.6 to 56.1% vs. 5.9% (placebo)	MDS-004 (Phase III) ^3^
**Deferasirox**	ICT	TD LR-MDS with iron overload	225	EFS on ICT: 3.9 years vs. 3 (placebo)	TELESTO (Phase II) ^4^
**Etrombopag, ** **Romiplostim**	TPO	LR-MDS with severe thrombocytopenia	169	PLT-R: 47% vs. 11% (placebo)	EQOL-MDS (Phase II) ^5^
**Luspatercept**	Erythroid maturation agents	TD LR-MDS, ESA-refractory ^6^	354; 229	RBC-TI: 58.5% vs. 31.2% (placebo); 38% vs. 13% (placebo)	MEDALIST (Phase III) ^6^, COMMANDS (Phase III) ^7^
**Azacitidine, ** **Decitabine, ** **Guadecitabine**	HMAs	TD LR-MDS, ESA-unresponsive	113	RBC-TI: 41% decitabine vs. 15% azacitadine	NCT01720225 (Phase II) ^8^
**Imetelstat**	Telomerase inhibitor	TD LR-MDS, ESA-refractory	178	RBC-TI: 39.8% vs. 15% (placebo)	IMerge (Phase III) ^9^
^1^ Inclusion: Hb ≤ 10.0 g/dL, ≤4 RBC units/8 weeks, serum EPO < 500 mU/mL.					
^2^ ESAs have been studied in multiple trials over decades (ECOG E1996 Trial, ARCADE Trial); no single definitive phase III trial established efficacy, though EPOANE3021 (NCT01381809) is one recent example showing ER of 45.9% vs. 4.4% at 24 weeks (*N* = 130).					
^3^ Inclusion: TD patients with del(5Q). Primary endpoint: RBC-TI ≥ 26 weeks. MDS-003 (phase II, *N* = 148) was the initial registration trial. NCT00179621.					
^4^ Inclusion: TD patients with serum ferritin > 2247 pmol/L (>1000 ng/mL) and prior receipt of 15–75 pRBCs. Primary endpoint: EFS from randomization to first nonfatal event (cardiac, hepatic, death, or AML transformation). Median EFS 1440 vs. 1091 days (*p* = 0.015). NCT00940602.					
^5^ Inclusion: Platelet count < 30 × 10^3^/mm^3^ with high bleeding risk. Primary endpoint: PLT response for ≥25 weeks. NCT02912208.					
^6^ COMMANDS (NCT03682536, *N* = 354): ESA-naive TD patients with or without ring sideroblasts; <5% blasts, sEPO < 500 U/L. Primary endpoint: RBC-TI ≥ 12 weeks with Hb increase ≥ 1.5 g/dL within the first 24 weeks. Compared luspatercept vs. epoetin alfa (first-line, head-to-head comparison). First drug to demonstrate superiority over ESAs in first-line treatment of LR-MDS.					
^7^ MEDALIST (NCT02631070, *N* = 229): Registration trial in ESA-refractory or failed patients with ring sideroblasts (≥15% RS or ≥5% with SF3B1 mutation); <5% blasts, sEPO ≤ 500 U/L. Primary endpoint: RBC-TI ≥ 8 weeks during weeks 1–24. Compared luspatercept vs. placebo. Led to initial FDA approval (2020) for ESA-refractory, RS+ disease.					
^8^ Inclusion: TD patients unresponsive to ESAs with refractory anemia and ringed sideroblasts. Primary endpoint: ORR at 8 weeks.					
^9^ Inclusion: TD patients relapsed, refractory, or ineligible for ESAs; non-del(5q); no prior lenalidomide or HMA. Primary endpoint: RBC-TI ≥ 8 weeks. Secondary endpoints included RBC-TI ≥ 24 weeks (28% vs. 3%). NCT02598661.					

Abbreviations: ER = erythroid response; ESA = erythropoiesis stimulating agent; RBC-TI = red blood cell transfusion independence; IMiDs = immunomodulatory drugs; EFS = event-free survival; ICT = iron chelation therapy; TPO = thrombopoietin; PLT-R = platelet response; HMAs = hypomethylating agents; ORR = overall response rate.

**Table 2:** Experimental therapies in pipeline for low-risk MDS.

Agent	Class/Target	Included patients	N	Status/Outcomes	Trial (Phase)
**Roxadustat**	HIF-PH inhibitors	LR-MDS, low transfusion burden ^1^	184	RBC-TI: 47.5% vs. 33.3% (placebo)	MATTERHORN (Phase-III) ^1^
**Oral azacitadine**	Hypomethylating agent	LR-MDS, low/moderate transfusion burden ^2^	216	RBC-TI: 31% vs. 11% (placebo)	AZA-MDS-003 (Phase III) ^2^
**K562/GM-CSF**	Whole cell cancer vaccine	LR-MDS, high transfusion burden ^3^	5	No serious AEs noted	Pilot trial ^3^
**Oral Decitabine** **and** **Cedazuridine**	DNMT inhibitors	LR-MDS, moderate transfusion burden ^4^	27	RBC-TI: 48%	NCT03502668 (Phase I/II) ^4^
**H3B-8800,** **E-7107**	Splicing modulator	TD LR-MDS, serum EPO > 500 mU/mL ^5^	42	RBC-TI: 19%	Encore-MDS (Phase I) ^5^
**R289**	IRAK1/2 inhibitor	LR-MDS refractory to EPO, TPO, luspatercept, or HMAs ^6^	Enrolling	RBC-TI/HI-E: 40% (≥500 mg QD)	NCT05308264 (Phase IB) ^6^
**Pexmetinib** **(ARRY-614)**	p38 MAPK/Tie2 Dual Inhibitor	LR-MDS, heavily pre-treated ^7^	44	HI: 32%	NCT00916227 (Phase I) ^7^
**Tebapivat** **(AG-946)**	Pyruvate kinase activator	LR-MDS, low transfusion burden, Hb < 11.0 ^8^	Enrolling	Currently enrolling	NCT05490446 (Phase I/II) ^8^
**Ezatiostat**	GSTP1-1 inhibitor	LR-MDS with or without thrombocytopenia ^9^	86	HI-E: 29%	NCT00700206 (Phase II) ^9^
**Divesiran** **(SLN124)**	siRNA targeting TMPRSS6	LR-MDS with ferritin > 250 ng/mL ^10^	44	No serious TEAEs	NCT04718844 (Phase I/II) ^10^
**Emavusertib**	IRAK4 inhibitor	LR-MDS with cytopenia, ESA-naive or refractory ^11^	36	Study terminated	LUCAS (Phase II) ^11^
**Tomaralimab**	Toll-like Receptor 2 blocker	LR-MDS, heavy transfusion burden, heavily pre-treated ^12^	51	ORR: 50%	NCT02363491 (Phase I/II) ^12^
**Itacnosertib**	ACVR1/ALK5 inhibitor	LR-MDS ESA-refractory, 5q deletion, low/high transfusion burden ^13^	N/A	Study terminated	NCT04623996 (Phase I/II) ^13^
**Elritercept**	FLT3-ALK2 dual inhibitor	LR-MDS, low transfusion burden ^14^	59	TEAEs: 32%	NCT04419649 (Phase II) ^14^
**Vactosertib**	TGF- β receptor type-1 inhibitor	LR-MDS TD, PLT < 100, del-5q ^15^	9	No published outcomes	NCT03074006 (Phase I/II) ^15^
**Canakinumab**	IL-1β inhibitor	LR-MDS refractory to ≥1 line ^16^	23	No serious TEAEs	NCT04239157 (Phase II) ^16^
**BI 836858**	Anti-CD33 monoclonal antibody	LR-MDS with symptomatic anemia, with/without del-5q ^17^	27	TEAEs: 12%	NCT02240706 (Phase I/II) ^17^
**Rigosertib**	RAS effector pathway inhibitor	LR-MDS TD, ESA-refractory ^18^	62	TI: 32%	NCT01904682 (Phase II) ^18^
^1^ Inclusion: LR-MDS patients with low transfusion burden. Primary endpoint: RBC-TI × 8 weeks. Roxadustat is an oral HIF-PH inhibitor. HORN trial (NCT02263091).					
^2^ Inclusion: LR-MDS with low/moderate transfusion burden. Primary endpoint: RBC-TI × 8 weeks. Oral azacitidine formulation for outpatient use. AZA-MDS-003 trial (NCT01566695).					
^3^ Inclusion: LR-MDS with high transfusion burden. Primary endpoint: Dose-limiting toxicity. Whole cell cancer vaccine pilot study.					
^4^ Inclusion: LR-MDS with moderate transfusion burden, PLT <50, Hb <9.0. Primary endpoint: Dose-limiting toxicity and HI × 18–24 months. Oral formulation combining decitabine with cedazuridine (cytidine deaminase inhibitor).					
^5^ Inclusion: TD LR-MDS with serum EPO > 500 mU/mL, PLT > 50, collapsed/relapsed after EPO. Primary endpoint: Dose escalation and dose-limiting toxicity. H3B-8800 is an oral SF3B1 splicing modulator. Phase I trial (Encore-MDS) completed: 19% RBC-TI rate in 42 patients, with 5 of 15 SF3B1-mutant MDS patients achieving RBC-TI. E-7107 development was discontinued due to toxicity. H3B-8800 development appears to have been discontinued (NCT02841540).					
^6^ Inclusion: LR-MDS refractory to EPO, TPO-agents, luspatercept, or HMAs. Primary endpoint: Safety and tolerability. R289 is a prodrug of R835, a potent and selective dual IRAK1/4 inhibitor. Received FDA Fast Track Designation (December 2024) and Orphan Drug Designation (January 2025) for previously treated transfusion-dependent LR-MDS. Preliminary phase IB data [95]: 40% RBC-TI/HI-E responses at doses ≥ 500 mg QD in heavily pretreated patients; well-tolerated with mostly grade 1–2 adverse events (NCT05308264).					
^7^ Inclusion: LR-MDS, heavily pre-treated patients. Primary endpoint: Dose-escalation. Pexmetinib (formerly ARRY-614) is a dual p38 MAPK/Tie2 inhibitor. Phase I trial showing 32% hematologic improvement. No MTD reached for once-daily dosing; 1200 mg QD recommended for further study. Most common AEs were rash, diarrhea, dry skin, and fatigue (NCT00916227).					
^8^ Inclusion: LR-MDS with low transfusion burden, Hb < 11.0. Primary endpoint: Hemoglobin response × 16 weeks. Pyruvate kinase activator. Trial currently enrolling (NCT05490446).					
^9^ Inclusion: LR-MDS with anemia with or without thrombocytopenia. Primary endpoint: HI-E × 24 weeks. GSTP1-1 inhibitor showing 29% HI-E rate (NCT00700206).					
^10^ Inclusion: LR-MDS with ferritin > 250 ng/mL, Hb < 11.0 g/dL. Primary endpoint: Dose-limiting toxicities. Divesiran (formerly SLN124) is a GalNAc-siRNA targeting TMPRSS6 to increase hepcidin and reduce iron overload. Currently enrolling in phase I/II for MDS. Has Orphan Drug Designation for MDS, thalassemia, and polycythemia vera. No serious TEAEs reported in healthy volunteer and patient studies (NCT04718844).					
^11^ Inclusion: LR-MDS with cytopenia, ESA-naive or refractory. Primary endpoint: HI-E × 16 weeks. IRAK4 inhibitor. Study terminated. LUCAS trial (NCT05178342).					
^12^ Inclusion: LR-MDS with heavy transfusion burden, heavily pre-treated. Primary endpoint: Dose-limiting toxicities. TLR2 blocker showing 50% ORR (NCT02363491).					
^13^ Inclusion: LR-MDS ESA-refractory, 5q deletion, and low or high transfusion burden. Primary endpoint: Dose-limiting toxicities. ACVR1/ALK5 inhibitor. Study terminated (NCT04623996).					
^14^ Inclusion: LR-MDS with low transfusion burden. Primary endpoint: Dose-limiting toxicities. Elritercept (also called KER-050) is a second-generation TGF-β modulator, 32% experiencing TEAEs (NCT04419649).					
^15^ Inclusion: LR-MDS with transfusion dependence, PLT < 100, del-5q. Primary endpoint: Dose-limiting toxicities, HI. TGF-β receptor type-1 inhibitor. No published outcomes (NCT03074006).					
^16^ Inclusion: LR-MDS refractory to ≥1 line of treatment. Primary endpoint: Dose-limiting toxicities. IL-1β inhibitor. No serious TEAEs reported (NCT04239157).					
^17^ Inclusion: LR-MDS with symptomatic anemia with or without del-5q. Primary endpoint: Dose-limiting toxicities. BI 836,858 is a fully human, Fc-engineered anti-CD33 monoclonal antibody designed for enhanced antibody-dependent cellular cytotoxicity (ADCC). A percentage of 12% TEAEs, has Orphan Drug Designation for MDS (NCT02240706).					
^18^ Inclusion: LR-MDS with transfusion dependence and ESA-refractory. Primary endpoint: TI × 24 weeks. RAS effector pathway inhibitor showing 32% TI rate (NCT01904682).					

Abbreviations: HIF-PH = hypoxia-inducible factor prolyl hydroxylase; RBC-TI = red blood cell transfusion independence; LR-MDS = low-risk myelodysplastic syndrome; HMAs = hypomethylating agents; DNMT = DNA methyltransferase; TD = transfusion dependent; EPO = erythropoietin; TPO = thrombopoietin; IRAK = interleukin-1 receptor-associated kinase; HI = hematologic improvement; HI-E = hematologic improvement-erythroid; GSTP1-1 = glutathione S-transferase P1-1; siRNA = small interfering RNA; TMPRSS6 = transmembrane serine protease 6; GalNAc = N-acetylgalactosamine; TEAEs = treatment-emergent adverse events; ESA = erythropoiesis-stimulating agent; TLR2 = toll-like receptor 2; ORR = overall response rate; ACVR1 = activin A receptor type 1; ALK5 = activin receptor-like kinase 5; FLT3 = FMS-like tyrosine kinase 3; ALK2 = activin receptor-like kinase 2; TGF-β = transforming growth factor beta; PLT = platelet; TI = transfusion independence; MAPK, mitogen-activated protein kinase; QD, once daily.

## Data Availability

No new data were created or analyzed in this study. Data sharing is not applicable to this article.
