# Correction: Dhillon et al. Molecular Insights and Therapeutic Advances in Low-Risk Myelodysplastic Neoplasms: A Clinical Review. *Cancers* 2025, *17*, 3610

**DOI:** 10.3390/cancers18010172

**Published:** 2026-01-04

**Authors:** Vikram Dhillon, Jaroslaw Maciejewski, Suresh Kumar Balasubramanian

**Affiliations:** 1 Department of Oncology, Karmanos Cancer Institute, Wayne State University, Detroit, MI 48201, USA; hf9375@wayne.edu; 2 Department of Translational Hematology and Oncology Research, Taussig Cancer Institute, Cleveland Clinic Foundation, Cleveland, OH 44195, USA; maciejj@ccf.org

## Error in Figure 1 Legend

In the original publication [1], there was an error in the legend for Figure 1 as published. An incorrect version of the Figure 1 legend was inadvertently included in the revised manuscript. The correct legend appears below.

**Figure 1.** Diagnostic algorithm for pre-MDS conditions. This flowchart illustrates the diagnostic pathway for distinguishing between clonal and non-clonal cytopenias and dysplasia that can precede or may progress to MDS. The algorithm differentiates four key entities based on the presence or absence of cytopenias, dysplastic features, and clonal mutations: ICUS (Idiopathic Cytopenias of Uncertain Significance)—cytopenia without clonal mutations or dysplasia; CCUS (Clonal Cytopenias of Uncertain Significance)—cytopenia with clonal mutations but without dysplasia; CHIP (Clonal Hematopoiesis of Indeterminate Potential)—clonal mutations without cytopenia or dysplasia; and IDUS (Idiopathic Dysplasia of Unknown Significance)—morphologic dysplasia without cytopenia or clonal mutations. ^#^ High risk mutations include *ASXL1*, *CBL*, *DNMT3A*, *ETV6*, *EZH2*, *IDH2*, *KRAS*, *NPM1*, *NRAS*, *RUNX1*, *SF3B1*, *SRSF2*, and *U2AF1*.

## Error in Figure 1

In the original publication, there was an error in Figure 1 as published. An incorrect version of Figure 1 was inadvertently included in the revised manuscript. The corrected Figure 1 appears below.

## Error in Figure 3 Legend

In the original publication, there was an error in the legend for Figure 3 as published. An incorrect version of the Figure 3 legend was inadvertently included in the revised manuscript. The correct legend appears below.

**Figure 3.** Our treatment approach to low-risk myelodysplastic syndrome (LR-MDS). This flowchart outlines the therapeutic approach to low-risk MDS based on clinical presentation and laboratory parameters. The algorithm stratifies patients into four categories: Moderate and asymptomatic cytopenias (observation only), symptomatic anemia, symptomatic neutropenia, and symptomatic thrombocytopenia. Green boxes with solid backgrounds indicate FDA-approved therapies; green boxes with italicized text denote clinically used but not formally approved treatments. Blue boxes represent clinical decision points. Abbreviations: ATG, antithymocyte globulin; del(5q), deletion of chromosome 5q; chr, chromosome; EPO, erythropoietin; G-CSF, granulocyte colony-stimulating factor; Hb, hemoglobin; MDS, myelodysplastic syndrome; RBC, red blood cell; RS, ring sideroblasts; SF3B1, splicing factor 3B subunit 1 gene; TPO-RAs, thrombopoietin receptor agonists; and U/L, units per liter.

## Error in Figure 3

In the original publication, there was an error in Figure 3 as published. An incorrect version of Figure 3 was inadvertently included in the revised manuscript. The corrected Figure 3 appears below.

## Error in Table 1

In the original publication, there was an error in Table 1 as published. Under Imetelstat efficacy outcomes, the RBC-TI percentage was incorrectly listed as 40% when it should be 39.8%. The corrected Table 1 appears below.

## Text Correction

There was an error in the original publication. Under Section 3.6. Telomerase inhibitor: Imetelstat, the RBC-TI percentage was incorrectly listed as 40% when it should be 39.8%. A correction has been made to Section 3.6, second paragraph:

Follow-up phase-II studies demonstrated durable transfusion independence in 42% of LR-MDS patients ineligible for ESA therapy [48]. The Phase-III IMerge trial (NCT02598661) enrolled 178 patients randomized to imetelstat or placebo [49]. In the treatment arm, about 40% achieved RBC-transfusion independence with durable responses lasting 52 weeks. Molecular profiling revealed *SF3B1* as the most frequently mutated gene (75.8%), with *SF3B1*-mutant patients demonstrating superior transfusion independence rates with Imetelstat versus placebo (48.8% vs. 16.3% at 8 weeks) [49]. Responses varied across *SF3B1* hotspots, with T663P and A744P mutations achieving 100% response rates, while the most common K700E hotspot achieved 43.9% response. Other frequently mutated genes also responded: *TET2*-mutant patients achieved 50% transfusion independence versus 21.4% with placebo, and *ASXL1*-mutant patients achieved 27.8% response versus 0% with placebo [49]. Notably, higher mutational burden (>2 mutations) was associated with enhanced response rates (45.5% versus 6.7% with placebo), and even patients harboring traditionally poor-prognosis mutations (*TP53*, *ETV6*, *RUNX1*, *ASXL1*, or *EZH2*) achieved 31.8% transfusion independence with Imetelstat versus 0% with placebo [50]. Neutropenia was the most serious adverse event (91% treatment arm vs. 47% placebo), though it is manageable with dose delays and reductions, and based on this trial, Imetelstat has received FDA approval for LR-MDS in heavily TD patients.

The authors state that the scientific conclusions are unaffected. This correction was approved by the Academic Editor. The original publication has also been updated.

## Figures and Tables

**Figure 1:**
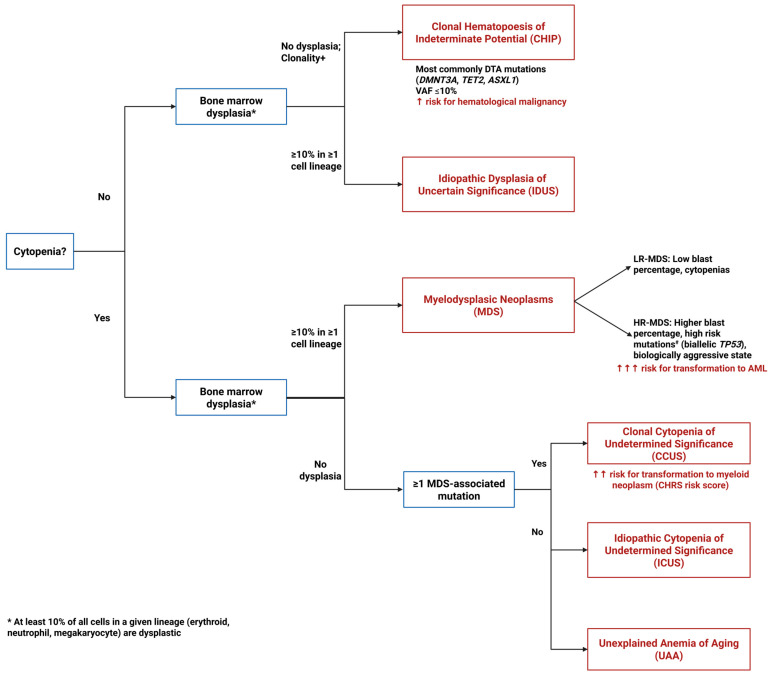
Diagnostic algorithm for pre-MDS conditions. This flowchart illustrates the diagnostic pathway for distinguishing between clonal and non-clonal cytopenias and dysplasia that can precede or may progress to MDS. The algorithm differentiates four key entities based on the presence or absence of cytopenias, dysplastic features, and clonal mutations: ICUS (Idiopathic Cytopenias of Uncertain Significance)—cytopenia without clonal mutations or dysplasia; CCUS (Clonal Cytopenias of Uncertain Significance)—cytopenia with clonal mutations but without dysplasia; CHIP (Clonal Hematopoiesis of Indeterminate Potential)—clonal mutations without cytopenia or dysplasia; and IDUS (Idiopathic Dysplasia of Unknown Significance)—morphologic dysplasia without cytopenia or clonal mutations. ^#^ High risk mutations include *ASXL1*, *CBL*, *DNMT3A*, *ETV6*, *EZH2*, *IDH2*, *KRAS*, *NPM1*, *NRAS*, *RUNX1*, *SF3B1*, *SRSF2*, and *U2AF1*.

**Figure 3:**
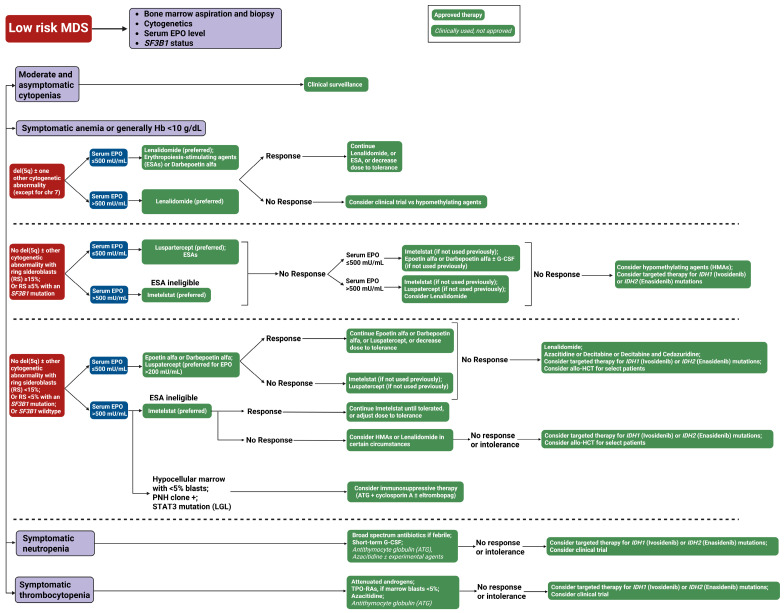
Our treatment approach to low-risk myelodysplastic syndrome (LR-MDS). This flowchart outlines the therapeutic approach to low-risk MDS based on clinical presentation and laboratory parameters. The algorithm stratifies patients into four categories: Moderate and asymptomatic cytopenias (observation only), symptomatic anemia, symptomatic neutropenia, and symptomatic thrombocytopenia. Green boxes with solid backgrounds indicate FDA-approved therapies; green boxes with italicized text denote clinically used but not formally approved treatments. Blue boxes represent clinical decision points. Abbreviations: ATG, antithymocyte globulin; del(5q), deletion of chromosome 5q; chr, chromosome; EPO, erythropoietin; G-CSF, granulocyte colony-stimulating factor; Hb, hemoglobin; MDS, myelodysplastic syndrome; RBC, red blood cell; RS, ring sideroblasts; SF3B1, splicing factor 3B subunit 1 gene; TPO-RAs, thrombopoietin receptor agonists; and U/L, units per liter.

**Table 1:** Currently approved for low-risk MDS.

Agent	Class/Target	Included Patients	*N*	Efficacy Outcomes	Trial (Phase)
**Erythropoietin alpha,** **Darbepoetin alpha**	ESA	LR-MDS patients with anemia, low transfusion burden ^1^	130	ER: 45.9% vs. 4.4% (placebo)	EPOANE3021 (Phase III) ^2^
**Lenalidomide**	IMiDs	TD LR-MDS with del (5Q)	205	RBC-TI: 42.6 to 56.1% vs. 5.9% (placebo)	MDS-004 (Phase III) ^3^
**Deferasirox**	ICT	TD LR-MDS with iron overload	225	EFS on ICT: 3.9 years vs. 3 (placebo)	TELESTO (Phase II) ^4^
**Etrombopag, ** **Romiplostim**	TPO	LR-MDS with severe thrombocytopenia	169	PLT-R: 47% vs. 11% (placebo)	EQOL-MDS (Phase II) ^5^
**Luspatercept**	Erythroid maturation agents	TD LR-MDS, ESA-refractory ^6^	354; 229	RBC-TI: 58.5% vs. 31.2% (placebo); 38% vs. 13% (placebo)	MEDALIST (Phase III) ^6^, COMMANDS (Phase III) ^7^
**Azacitidine, ** **Decitabine, ** **Guadecitabine**	HMAs	TD LR-MDS, ESA-unresponsive	113	RBC-TI: 41% decitabine vs. 15% azacitadine	NCT01720225 (Phase II) ^8^
**Imetelstat**	Telomerase inhibitor	TD LR-MDS, ESA-refractory	178	RBC-TI: 39.8% vs. 15% (placebo)	IMerge (Phase III) ^9^
^1^ Inclusion: Hb ≤ 10.0 g/dL, ≤4 RBC units/8 weeks, serum EPO < 500 mU/mL.					
^2^ ESAs have been studied in multiple trials over decades (ECOG E1996 Trial, ARCADE Trial); no single definitive phase III trial established efficacy, though EPOANE3021 (NCT01381809) is one recent example showing ER of 45.9% vs. 4.4% at 24 weeks (*N* = 130).					
^3^ Inclusion: TD patients with del(5Q). Primary endpoint: RBC-TI ≥ 26 weeks. MDS-003 (phase II, *N* = 148) was the initial registration trial. NCT00179621.					
^4^ Inclusion: TD patients with serum ferritin > 2247 pmol/L (>1000 ng/mL) and prior receipt of 15–75 pRBCs. Primary endpoint: EFS from randomization to first nonfatal event (cardiac, hepatic, death, or AML transformation). Median EFS 1440 vs. 1091 days (*p* = 0.015). NCT00940602.					
^5^ Inclusion: Platelet count < 30 × 10^3^/mm^3^ with high bleeding risk. Primary endpoint: PLT response for ≥25 weeks. NCT02912208.					
^6^ COMMANDS (NCT03682536, *N* = 354): ESA-naive TD patients with or without ring sideroblasts; <5% blasts, sEPO < 500 U/L. Primary endpoint: RBC-TI ≥ 12 weeks with Hb increase ≥ 1.5 g/dL within the first 24 weeks. Compared luspatercept vs. epoetin alfa (first-line, head-to-head comparison). First drug to demonstrate superiority over ESAs in first-line treatment of LR-MDS.					
^7^ MEDALIST (NCT02631070, *N* = 229): Registration trial in ESA-refractory or failed patients with ring sideroblasts (≥15% RS or ≥5% with SF3B1 mutation); <5% blasts, sEPO ≤ 500 U/L. Primary endpoint: RBC-TI ≥ 8 weeks during weeks 1–24. Compared luspatercept vs. placebo. Led to initial FDA approval (2020) for ESA-refractory, RS+ disease.					
^8^ Inclusion: TD patients unresponsive to ESAs with refractory anemia and ringed sideroblasts. Primary endpoint: ORR at 8 weeks.					
^9^ Inclusion: TD patients relapsed, refractory, or ineligible for ESAs; non-del(5q); no prior lenalidomide or HMA. Primary endpoint: RBC-TI ≥ 8 weeks. Secondary endpoints included RBC-TI ≥ 24 weeks (28% vs. 3%). NCT02598661.					

Abbreviations: ER = erythroid response; ESA = erythropoiesis stimulating agent; RBC-TI = red blood cell transfusion independence; IMiDs = immunomodulatory drugs; EFS = event-free survival; ICT = iron chelation therapy; TPO = thrombopoietin; PLT-R = platelet response; HMAs = hypomethylating agents; ORR = overall response rate.
